# Epidemiologic and Genomic Analysis of SARS-CoV-2 Delta Variant Superspreading Event in Nightclub, the Netherlands, June 2021

**DOI:** 10.3201/eid2805.212019

**Published:** 2022-05

**Authors:** Jelle Koopsen, Catharina E. van Ewijk, Roisin Bavalia, Akke Cornelissen, Sylvia M. Bruisten, Floor de Gee, Alvin X. Han, Maarten de Jong, Menno D. de Jong, Marcel Jonges, Norin Khawaja, Fleur M.H.P.A. Koene, Mariken van der Lubben, Iris Mikulic, Sjoerd P.H. Rebers, Colin A. Russell, Janke Schinkel, Anja J.M. Schreijer, Judith A. den Uil, Matthijs R.A. Welkers, Tjalling Leenstra, on behalf of the ARGOS consortium

**Affiliations:** Amsterdam University Medical Centres, University of Amsterdam, Amsterdam, the Netherlands (J. Koopsen, A.X. Han, M.D. de Jong, M. Jonges, S.P.H. Rebers, C.A. Russell, J. Schinkel, M.R.A. Welkers);; Public Health Service Amsterdam, the Netherlands. (C.E. van Ewijk, R. Bavalia, A. Cornelissen, S.M. Bruisten, F. de Gee, M. de Jong, N. Khawaja, F.M.H.P.A. Koene, M. van der Lubben, I. Mikulic, A.J.M. Schreijer, J.A. den Uil, M.R.A. Welkers, T. Leenstra)

**Keywords:** COVID-19, coronavirus disease, SARS-CoV-2, severe acute respiratory syndrome coronavirus 2, viruses, respiratory infections, zoonoses, superspreading, outbreaks, phylogenetics, Amsterdam, the Netherlands

## Abstract

We report a severe acute respiratory syndrome coronavirus 2 superspreading event in the Netherlands after distancing rules were lifted in nightclubs, despite requiring a negative test or vaccination. This occurrence illustrates the potential for rapid dissemination of variants in largely unvaccinated populations under such conditions. We detected subsequent community transmission of this strain.

Because of decreasing severe acute respiratory syndrome coronavirus 2 (SARS-CoV-2) incidence rates in the Netherlands at the time, the government of the Netherlands lifted most restrictions on June 26, 2021 (week 25) (*1*). The mandate to stay at home and get tested if experiencing symptoms remained. However, wearing of facemasks was no longer mandatory if a distance of >1.5 meters could be maintained. Event attendees who were fully vaccinated or had tested negative for SARS-CoV-2 within the previous 40 hours (testing-for-access) did not have to wear facemasks or maintain 1.5-meter physical distancing. Persons meeting 1 of those criteria (tested or fully vaccinated) were given a QR code in the CoronaCheck application, commissioned by the government of the Netherlands (*2*), which allowed them access to events.

Shortly after June 26, coronavirus disease (COVID-19) cases surged in the greater Amsterdam region of the Netherlands (Appendix 1 Figure 1). Most infections were among young adults 18–30 years of age (Appendix 1 Figure 2), of whom only 14% were fully vaccinated at that time (*3*). A steep increase in reported clusters related to the hospitality sector, particularly bars and discotheques, was observed in the following weeks; 121 clusters were reported in week 27 compared with an average of 4 clusters/week in weeks 21–25 (Appendix 1 Figure 3). To gain insight into the case surge and transmission dynamics, we investigated an outbreak linked to a nightclub event in central Amsterdam on June 26. We examined whether the high number of cases linked to the nightclub were the result of a superspreading event or the attendance of multiple infectious persons.

## The Study

In the Netherlands, confirmed infections are reported to the local Public Health Service (PHS), and source and contact tracing is performed with a telephone interview. Data are obtained on sociodemographics, date of symptom onset, symptoms, vaccination status (and, if applicable, vaccine type, number of doses, and dates of administration) and locations the index-patient visited during the incubation and contagious periods. Medical ethics clearance for this study was not required (Appendix 1).

We defined a case as illness in a person who visited the nightclub on June 26, tested positive for SARS-CoV-2 within 14 days, and whose status was reported to the PHS. Cases were identified passively: persons were included only if they indicated during their PHS interview that they had visited the nightclub on June 26. The nightclub has an estimated capacity of 150 persons and was reported to be at full capacity that evening with attendees dancing and singing to loud music. A total of 60 confirmed COVID-19 cases were linked to the nightclub, raising suspicion of a superspreading event. Onset of symptoms occurred during June 27–July 3. Most case-patients were not fully vaccinated (defined as 14 days after completion of the vaccination series [Appendix 1 Figures 4, 5]): 4 (7.4%) persons were fully vaccinated and 41 (76%) were unvaccinated (Table). Most cases were in young adults (mean age 21.1 years [SD 3.3 years]) and women (60%), and most persons reported COVID-19–associated symptoms (93%). In 61% of cases, no other potential source for transmission besides the nightclub event was indicated. Of the 60 confirmed cases, 33 persons lived in the Amsterdam region and 27 resided in other regions (Table).

**Table:** Descriptive statistics of 60 persons with confirmed SARS-CoV-2 infection after nightclub event, Amsterdam, the Netherlands, June 2021*

Characteristics	No. (%)
Sex	
F	34 (60)
M	23 (40)
Unknown	3
Mean age, y (SD)	21.1 (3.3)
Unknown	3
Symptoms	
Symptomatic	55 (93)
Asymptomatic	4 (7)
Unknown	1
Vaccination status	
Fully vaccinated†	4 (7.4)
Incomplete vaccination	9 (17)
No vaccination received	41 (76)
Unknown	6
PHS Region	
PHS Amsterdam	33 (57)
Other PHS Region	25 (43)
Unknown	2
Other self-reported potential sources	
None: only nightclub on June 26th	35 (61)
Other hospitality sector	17 (30)
Education	1 (1.8)
Social gathering	2 (3.5)
Supermarket	1 (1.8)
Vaccination location	1 (1.8)
Unknown	3

*PHS, Public Health Service; SARS-CoV-2, severe acute respiratory syndrome coronavirus 2. †Fully vaccinated defined as 14 days after completion of vaccination series.

**Figure 1:**
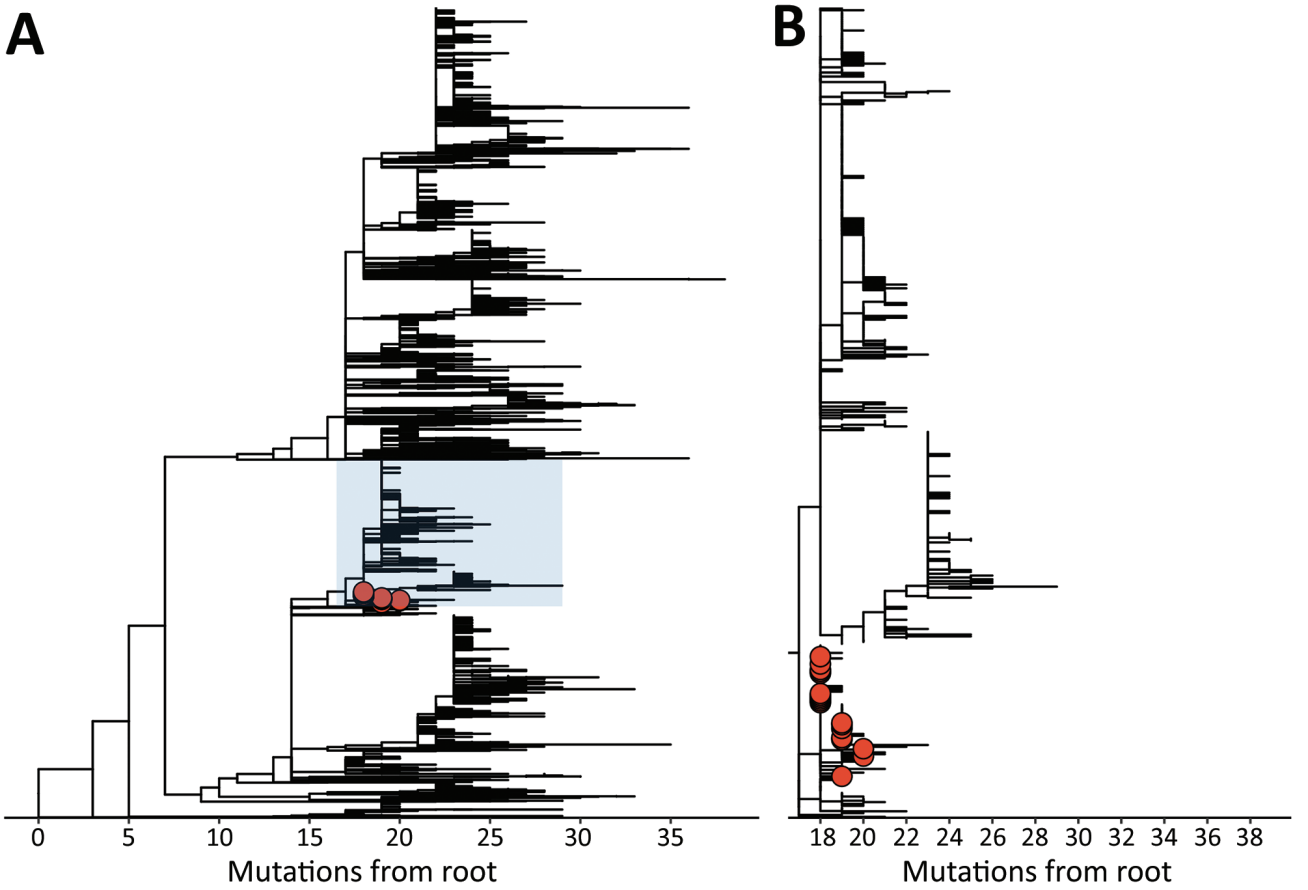
Clustering of severe acute respiratory syndrome coronavirus 2 cases related to nightclub event, Amsterdam, the Netherlands, June 2021. Red circles indicate sequences linked to the nightclub. A) Maximum-likelihood tree of all sequences (n = 4,905) in the dataset. B) Magnification of the clade (highlighted in blue in panel A) containing sequences linked to the nightclub (n = 1,663). Branches without tips depict other Netherlands Delta variant sequences derived from GISAID (https://www.gisaid.org).

Samples from 23/60 cases were available for sequencing, of which 3 were not eligible because of high cycle threshold values (>32). For 19/20 samples, we successfully obtained full genome sequences; all belonged to PANGO-lineage B.1.617.2 (*4*), which was denoted as variant of concern Delta by the World Health Organization (*5*) (Appendix 1 Table). To provide for phylogenetic context, we included weekly surveillance samples from the Amsterdam region (n = 421) in the analyses, as well as all Delta variant sequences from the Netherlands available in the GISAID database (https://www.gisaid.org; n = 4,465) (Appendix 2 Table) on August 1, 2021. All nightclub-associated genomes showed characteristics of a superspreading event: a tight phylogenetic cluster closely related in time (June 27–July 3) (Appendix 1 Figure 4) and genomic diversity (Figure 1). The pairwise genetic distance between all sequences was <2 single-nucleotide polymorphisms (Appendix 1 Figure 6), comparable to previously observed superspreading events (*6*). In addition, all sequences formed a monophyletic cluster marked by a specific single-nucleotide polymorphism combination: a Delta variant with C4321T in the presence of (wild-type) 22792C. In our dataset, all viruses with this combination collected before July 1 were sampled from persons who were at the nightclub. This combination was not observed in our dataset or in any Netherlands Delta sequences (n = 4465) from the GISAID database before June 26 (Appendix 1 Figure 7). Furthermore, randomly collected surveillance samples in the region from the weeks preceding the nightclub event showed diverse viruses circulating in the Amsterdam region (Appendix 1 Figure 8), and samples collected from 2 other nightclubs on June 26 also showed different lineages (Appendix 1 Figure 9). This finding makes multiple introductions at the nightclub with a highly prevalent, highly similar variant unlikely. In all, these findings strongly suggest a single introduction of the C4321T + 22792C variant, which was amplified by superspreading at the nightclub.

**Figure 2:**
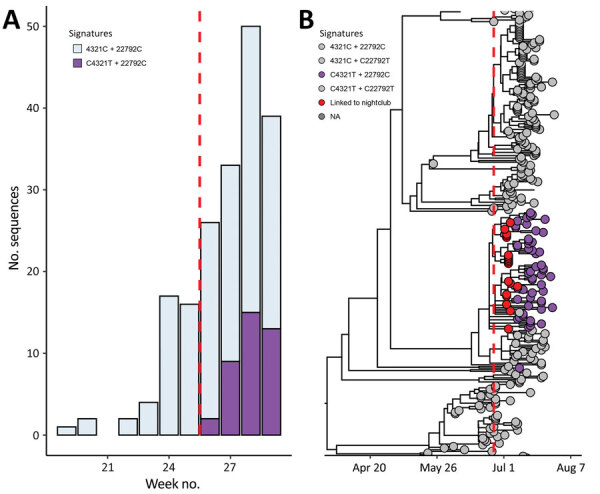
Detected increase of severe acute respiratory syndrome coronavirus 2 (SARS-CoV-2) sequences with signature first detected in nightclub samples, Amsterdam, the Netherlands. A) Absolute number of weekly cases in randomly selected surveillance samples in the Amsterdam region, colored by the nucleotides at position 4321 and 22792 of the SARS-CoV-2 genome. B) Time-resolved phylogenetic tree of dataset containing all Netherlands SARS-CoV-2 Delta variant sequences available in GISAID on August 1, 2021, random surveillance samples from the Amsterdam region, and samples from returning travelers to the Amsterdam region. Tips are colored by the nucleotides at position 4321 and 22792 and epidemiologic linkage to the nightclub (with signature C4321T + 22792C). Dashed red line indicates the day of lifting 1.5-meter social distancing restrictions with QR code. NA, not applicable.

Since the introduction of C4321T + 22792C, the variant has been increasingly detected in random genomic surveillance from the Amsterdam region: no surveillance samples were detected in week 26 compared with 33% of samples in week 28 (Figure 2). This lineage was introduced the weekend nightclubs were opened and has clearly propagated in the community, where subsequent transmission of the lineage occurred.

## Conclusions

This study illustrates the amplification of a specific linage in a largely unvaccinated group under circumstances such as those observed in a nightclub where social distancing measures and facemask requirements were lifted, despite a testing-for-access policy. In addition, our results highlight the consequence of superspreading events on subsequent transmission dynamics of SARS-CoV-2 in the community. Investigating an outbreak on June 26, 2021, the first date that social distancing measures were lifted under testing-for-access conditions, enabled us to isolate a single SARS-CoV-2 transmission event.

The role of superspreading in SARS-CoV-2 transmission has been highlighted previously (*6*,*7*), also in the context of nightclubs (*8*,*9*). Considering the potential of SARS-CoV-2 to be transmitted through aerosols (*10*,*11*), nightclubs can be a high-risk setting because of poor ventilation and sustained overcrowding. Our findings suggest that the rapid surge in cases in July 2021 was at least partially driven by superspreading events such as the event we describe.

In particular, testing-for-access, as it was put in place in the weeks following June 26, provided opportunity for infectious persons to slip through. Access was provided immediately after a single Johnson & Johnson/Janssen vaccination (https://www.janssen.com) (too soon), a negative antigen test result was valid for 40 hours (too long), and checking of QR codes was reported to be inconsistent at some venues (*12*,*13*).

This study used data collected for nonresearch purposes during scaled-down source and contact tracing and has limitations. First, cases were passively included, which could underestimate the true extent of the outbreak, because asymptomatic cases or cases tested only by self-administered antigen tests might have been missed. This factor could also explain the high percentage of symptomatic cases (*14*). Nevertheless, we believe this factor did not result in a biased selection of cases. Second, we conducted genomic analysis for only 1/121 detected hospitality sector–related clusters, limiting generalizability of our findings.

In conclusion, testing-for-access did not prevent superspreading at this event, indicating the need for caution when easing social distancing measures in night life, even under more optimal testing-for-access conditions. This finding is particularly relevant in a population where vaccination coverage is low or when new variants circulate that are associated with lower vaccine effectiveness.

Appendix 1 Additional information about epidemiologic and genomic analysis of SARS-CoV-2 Delta variant superspreading event in a nightclub, Amsterdam, the Netherlands, June 2021.

Appendix 2 Additional data used in epidemiologic and genomic analysis of SARS-CoV-2 Delta variant superspreading event in a nightclub, Amsterdam, the Netherlands, June 2021.
