# Supplementary material for: Epidemiologic and Genomic Analysis of SARS-CoV-2 Delta Variant Superspreading Event in Nightclub, the Netherlands, June 2021
Source: Emerg Infect Dis. 2022 May;28(5):1012–6. doi: 10.3201/eid2805.212019 (PMC9045423; doi:10.3201/eid2805.212019)
Supplement: Appendix 1 — Additional information about epidemiologic and genomic analysis of SARS-CoV-2 Delta variant superspreading event in a nightclub, Amsterdam, the Netherlands, June 2021. [file 21-2019-Techapp-s1.pdf]

Article DOI: https://doi.org/10.3201/eid2805.212019

Epidemiologic and Genomic Analysis of SARS-CoV-2 Delta Variant Superspreading Event in a Nightclub, the Netherlands, June 2021

Appendix

Supplementary Methods

Virus Detection and Whole-Genome Sequencing

Severe acute respiratory syndrome coronavirus 2 (SARS-CoV-2)–positive materials with cycle threshold values <32 were transported to the Department of Medical Microbiology of the Amsterdam University Medical Centre, location Meibergdreef, for full-genome sequencing. RNA extraction was performed on 200 μL of the original patient sample material by using a MagNaPure 96 System (Roche Diagnostics, https://www.roche.com) according to manufacturer's instructions with a total elution volume of 50 μL. Equine arteritis virus was added before the RNA extraction as an internal extraction control. For cDNA synthesis, the SuperScipt VILO cDNA Synthesis Kit (catalog no. 11754050, ThermoFisher Scientific, https://www.thermofisher.com) was used according to manufacturer's instructions. Extracted RNA was diluted to an estimated input of 100 copies/reaction in nuclease-free water (AM9939, Ambion, ThermoFisher Scientific). In total, 7 μL of diluted RNA solution was combined with 2 μL of 5xVILO reaction mix and 1 μL of 10x SuperScript III enzyme blend tot a total reaction volume of 10 μL. We performed cDNA synthesis on a 96-well Biometra thermal cycler for an initial step at 42°C for 30 minutes followed by 5 minutes at 85°C. We performed SARS-CoV-2 full-genome amplification, adaptor ligation, and purification by using the Ion AmpliSeq SARS-CoV-2 Insight Research Assay (A51305, ThermoFisher Scientific) according to manufacturer's instruction. Libraries were quantified by using the Ion Library TaqMan Quantitation Kit (4468802, ThermoFisher Scientific) according to manufacturer's instructions. Samples were sequenced on an Ion GeneStudio S5 system using an Ion 540 chip (ThermoFisher Scientific). Primer-removed fastq-files were exported for further analysis using the Torrent Suite Software (ThermoFisher Scientific).

Per read quality control was performed using Trimmomatic v0.36 (with settings "LEADING:20 TRAILING:20 SLIDINGWINDOW:10:20 MINLEN:50") (*1*). Resulting quality-checked reads were first mapped to human reference genome HG19 using BWA v0.7.17 (*2*) with default settings ("bwa bwasw") to remove all reads of potential human origin. All unmapped reads were subsequently mapped to SARS-CoV-2 refence genome Wuhan-Hu-1 (*3*). Resulting sequence alignment map (sam) files were converted to bam, sorted, and indexed using SAMtools v1.14 (*4*). A consensus sequence was generated using the TrueConsense package, kindly provided by the National Institute of Public Health and The Environment (RIVM) (sourcecode available at https://github.com/RIVM-bioinformatics/Trueconsense) using settings (-cov 10 -noambig).

Phylogenetic Analysis

Surveillance sequences are derived from randomly selected samples from the Amsterdam region to provide an indication of the circulating strains in the region. Returning travelers' surveillance sequences are derived from samples from returning travelers from countries deemed at risk for importation of variants of concern. A maximum-likelihood phylogeny and a time-resolved phylogeny were constructed using the Augur pipeline (*5*). We used procedures taken from [github.com/nextstrain/ncov] including the clock rate, reference genome, site masking, and clade definition files. More specifically, sequences were aligned to reference genome Wuhan-Hu-1 (*3*) using MAFFT (*6*), and a maximum-likelihood tree was constructed using IQ-TREE (*7*) under a HKY+G substitution model, while masking sites 13402, 24389, and 24390. The time-resolved tree was constructed using TreeTime (*8*) using a clock-rate of 0.0008 (SD 0.0004) and after removing molecular clock outliers (tips that deviate >4 interquartile ranges from the root-to-tip vs time regression). Trees were visualized using ggtree (*9*) as implemented in R (*10*). Sequences were deposited in GISAID (EPI_ISL3915592–EPI_ISL_3915610). Pairwise genetic distances were calculated with MEGA6 (*11*) using the distance matrix function with a nucleotide substitution p-distance model. P-distances were transformed to number of single-nucleotide polymorphisms, and are depicted in Appendix Figure 7. One single-nucleotide polymorphism was defined as a p-distance of 0.00003.

Ethics Statement

SARS-CoV-2 is a notifiable disease in the Netherlands. The Public Health Service has legal permission, provided by Dutch national public health law, to process patient information for national surveillance of communicable diseases (Wet Publieke Gezondheid,

https://wetten.overheid.nl/BWBR0024705/2021-07-17). Therefore, separate medical ethical clearance for this study was not required.

References

1. Bolger AM, Lohse M, Usadel B. Trimmomatic: a flexible trimmer for Illumina sequence data. Bioinformatics. 2014;30:2114–20. PubMed https://doi.org/10.1093/bioinformatics/btu170

2. Li H, Durbin R. Fast and accurate long-read alignment with Burrows-Wheeler transform. Bioinformatics. 2010;26:589–95. PubMed https://doi.org/10.1093/bioinformatics/btp698

3. Wu F, Zhao S, Yu B, Chen YM, Wang W, Song ZG, et al. A new coronavirus associated with human respiratory disease in China [Erratum in: Nature. 2020;580:E7]. Nature. 2020;579:265–9. PubMed https://doi.org/10.1038/s41586-020-2008-3

4. Li H, Handsaker B, Wysoker A, Fennell T, Ruan J, Homer N, et al.; 1000 Genome Project Data Processing Subgroup. The Sequence Alignment/Map format and SAMtools. Bioinformatics. 2009;25:2078–9. PubMed https://doi.org/10.1093/bioinformatics/btp352

5. Hadfield J, Megill C, Bell SM, Huddleston J, Potter B, Callender C, et al. Nextstrain: real-time tracking of pathogen evolution. Bioinformatics. 2018;34:4121–3. PubMed https://doi.org/10.1093/bioinformatics/bty407

6. Katoh K, Asimenos G, Toh H. Multiple alignment of DNA sequences with MAFFT. Methods Mol Biol. 2009;537:39–64. PubMed https://doi.org/10.1007/978-1-59745-251-9_3

7. Nguyen LT, Schmidt HA, von Haeseler A, Minh BQ. IQ-TREE: a fast and effective stochastic algorithm for estimating maximum-likelihood phylogenies. Mol Biol Evol. 2015;32:268–74. PubMed https://doi.org/10.1093/molbev/msu300

8. Sagulenko P, Puller V, Neher RA. TreeTime: Maximum-likelihood phylodynamic analysis. Virus Evol. 2018;4:vex042. PubMed https://doi.org/10.1093/ve/vex042

9. Yu G. Using ggtree to visualize data on tree-like structures. Curr Protoc Bioinformatics. 2020;69:e96. PubMed https://doi.org/10.1002/cpbi.96

10. R Core Team. R: A language and environment for statistical computing. Vienna, Austria: R Foundation for Statistical Computing; 2017.

11. Tamura K, Stecher G, Peterson D, Filipski A, Kumar S. MEGA6: Molecular Evolutionary Genetics Analysis version 6.0. Mol Biol Evol. 2013;30:2725–9. PubMed https://doi.org/10.1093/molbev/mst197

12. Ministry of General Affairs, the Netherlands. The Netherlands takes a big step: almost everything is possible at 1.5 meters [in Dutch]. 2021 Jun 18 [cited 2021 Aug 1]. https://www.rijksoverheid.nl/actueel/nieuws/2021/06/18/nederland-zet-grote-stap-bijna-alles-kan-op-anderhalve-meter

13. Ministry of General Affairs, the Netherlands. No choice but to take summertime measures in face of rapid increase in infections [in Dutch]. 2021 Jul 9 [cited 2021 Aug 1]. https://www.government.nl/latest/news/2021/07/09/no-choice-but-to-take-summertime-measures-in-face-of-rapid-increase-in-infections

Appendix Table. Sequence characteristics of SARS-CoV-2 infections linked to nightclub event, Amsterdam, the Netherlands, June 2021*

Sequence	Genome coverage†	Pango lineage	Variant	C_t value	GISAID strain
AUMC2021AC10	99.50%	B.1.617.2	Delta	18.90	hCoV-19/Netherlands/NH-AUMC-004568/2021
AUMC2021AC17	99.53%	B.1.617.2	Delta	21.58	hCoV-19/Netherlands/NH-AUMC-004575/2021
AUMC2021AC18	99.09%	B.1.617.2	Delta	24.23	hCoV-19/Netherlands/NH-AUMC-004595/2021
AUMC2021AC23	99.54%	B.1.617.2	Delta	25.72	hCoV-19/Netherlands/NH-AUMC-004536/2021
AUMC2021AC24	99.65%	B.1.617.2	Delta	25.17	hCoV-19/Netherlands/NH-AUMC-004531/2021
AUMC2021AC39	0%	NA	NA	25.57	Failed sequence
AUMC2021AC64	99.41%	B.1.617.2	Delta	27.98	hCoV-19/Netherlands/NH-AUMC-004572/2021
AUMC2021AC66	99.19%	B.1.617.2	Delta	29.81	hCoV-19/Netherlands/NH-AUMC-004576/2021
AUMC2021AD18	98.73%	B.1.617.2	Delta	17.16	hCoV-19/Netherlands/NH-AUMC-004595/2021
AUMC2021AD19	99.60%	B.1.617.2	Delta	17.81	hCoV-19/Netherlands/NH-AUMC-004594/2021
AUMC2021AD65	97.59%	B.1.617.2	Delta	26.22	hCoV-19/Netherlands/NH-AUMC-004586/2021
AUMC2021AE31	99.65%	B.1.617.2	Delta	20.32	hCoV-19/Netherlands/NH-AUMC-009904/2021
AUMC2021AE37	99.54%	B.1.617.2	Delta	20.08	hCoV-19/Netherlands/NH-AUMC-009926/2021
AUMC2021AE38	99.67%	B.1.617.2	Delta	19.44	hCoV-19/Netherlands/NH-AUMC-009535/2021
AUMC2021AE49	99.44%	B.1.617.2	Delta	23.07	hCoV-19/Netherlands/NH-AUMC-009880/2021
AUMC2021AE70	99.41%	B.1.617.2	Delta	24.70	hCoV-19/Netherlands/NH-AUMC-009927/2021
AUMC2021AE88	99.46%	B.1.617.2	Delta	31.48	hCoV-19/Netherlands/NH-AUMC-009536/2021
AUMC2021AE89	99.67%	B.1.617.2	Delta	28.75	hCoV-19/Netherlands/NH-AUMC-009534/2021
AUMC2021AE90	99.39%	B.1.617.2	Delta	28.01	hCoV-19/Netherlands/NH-AUMC-009925/2021
AUMC2021AF28	99.59%	B.1.617.2	Delta	22.70	hCoV-19/Netherlands/NH-AUMC-009542/2021

*C_t, cycle threshold; SARS-CoV-2, severe acute respiratory syndrome coronavirus 2.
†Genome coverage was calculated based on the length of the reference sequence MN908947 (Wuhan-Hu-1) (*3*).

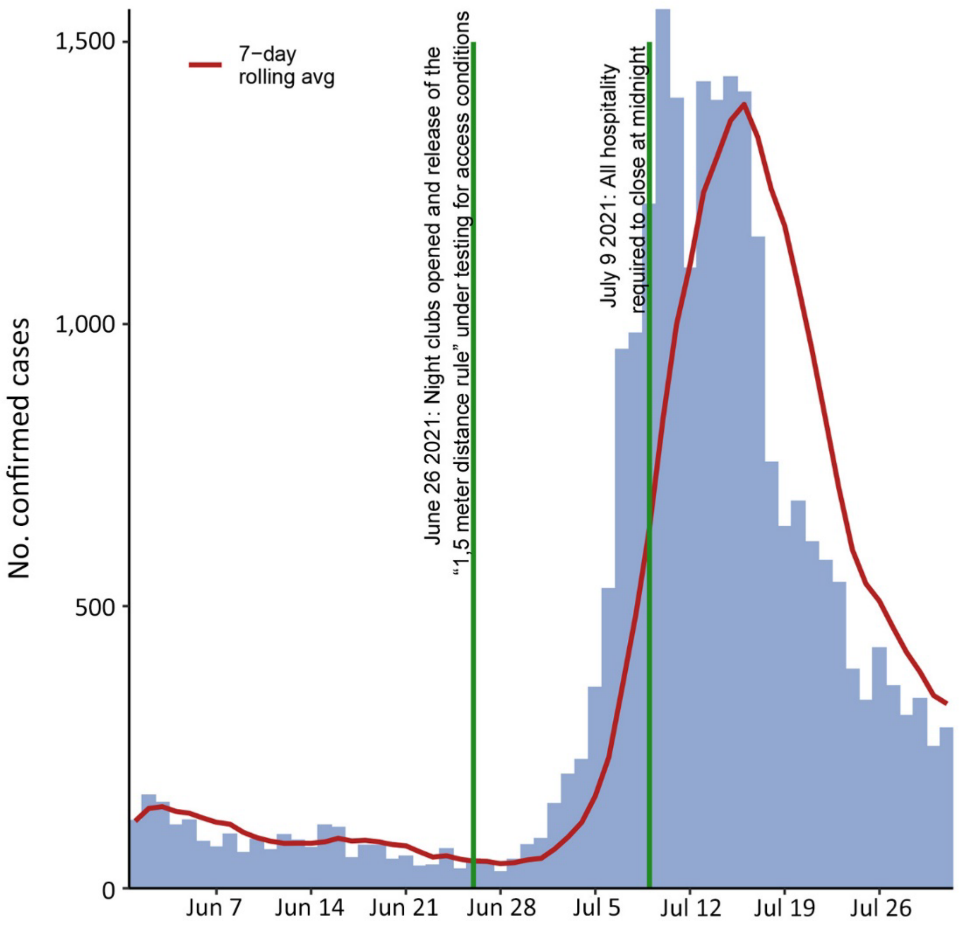

Appendix Figure 1. Epicurve of coronavirus disease cases in region of the municipal health service of Amsterdam, the Netherlands. Red line is 7-day rolling average. Green lines indicate dates of measure easing (*12*) and subsequent tightening (*13*).

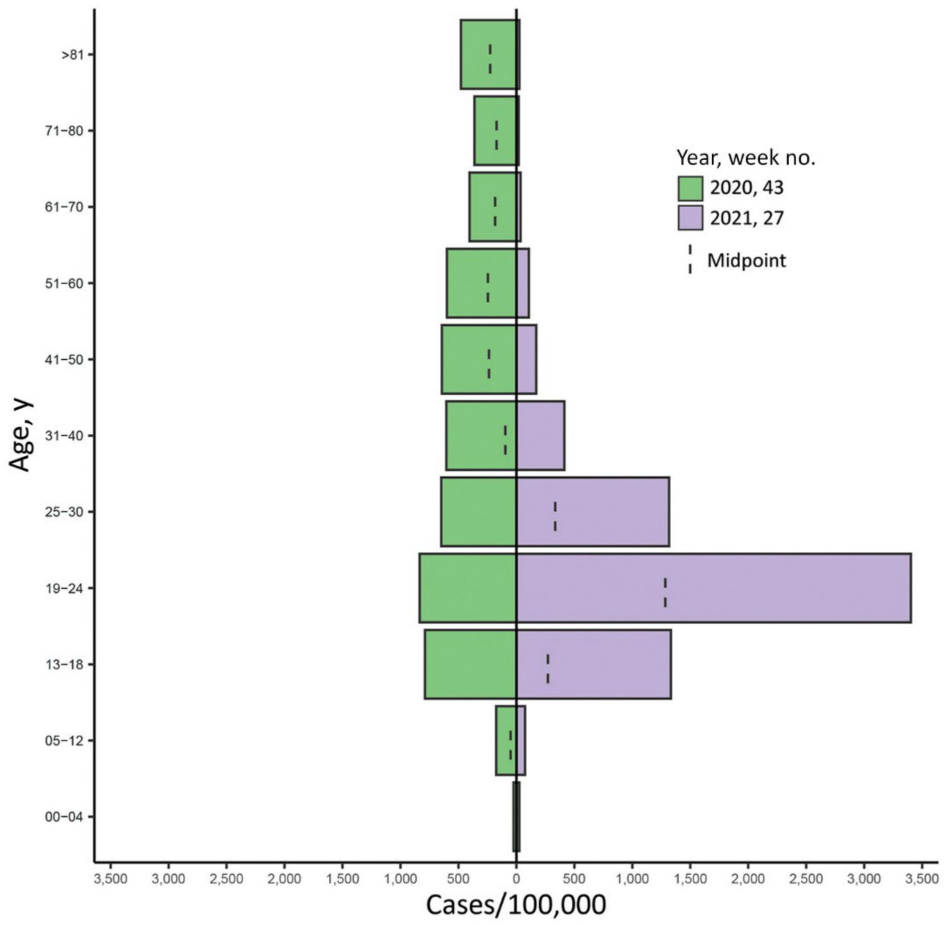

Appendix Figure 2. Coronavirus disease cases per 100,000 persons in the Amsterdam region in 2 periods of rapid increase in cases, the Netherlands. Age distributions of all cases in the Amsterdam region in week 43 in 2020 (Autumn 2020 increase) are compared with age distributions of all cases in the Amsterdam region in week 27 in 2021 (most recent increase).

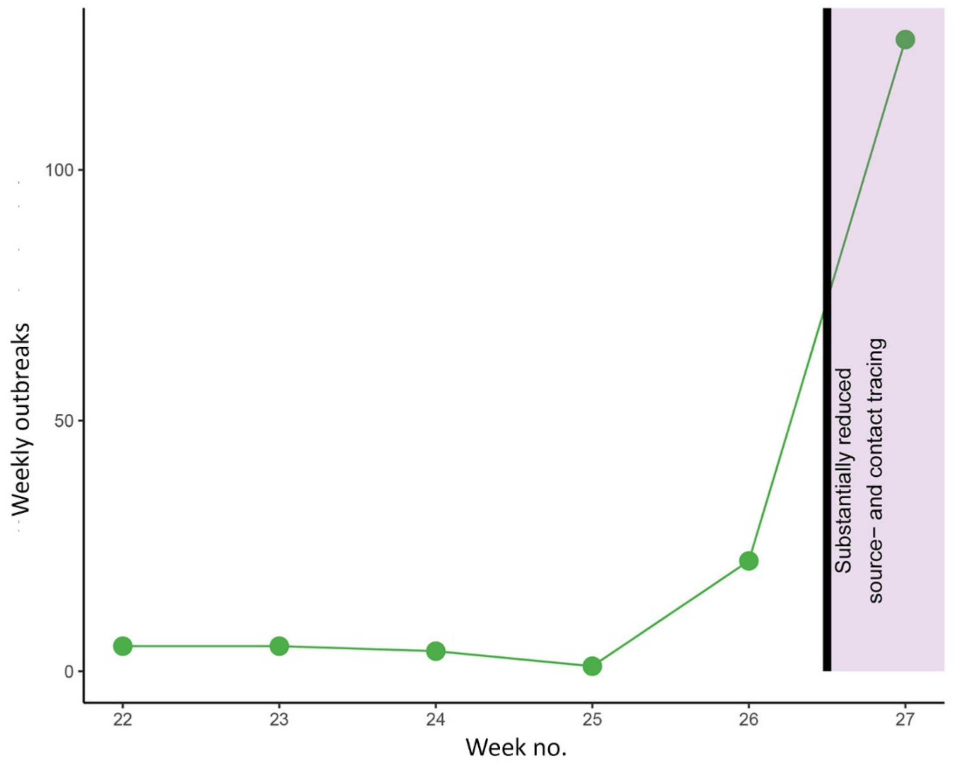

Appendix Figure 3. Number of weekly coronavirus disease cases related to hospitality in the Amsterdam region, the Netherlands. Shaded area indicates a period of scaled-down source and contact tracing, indicating underreporting.

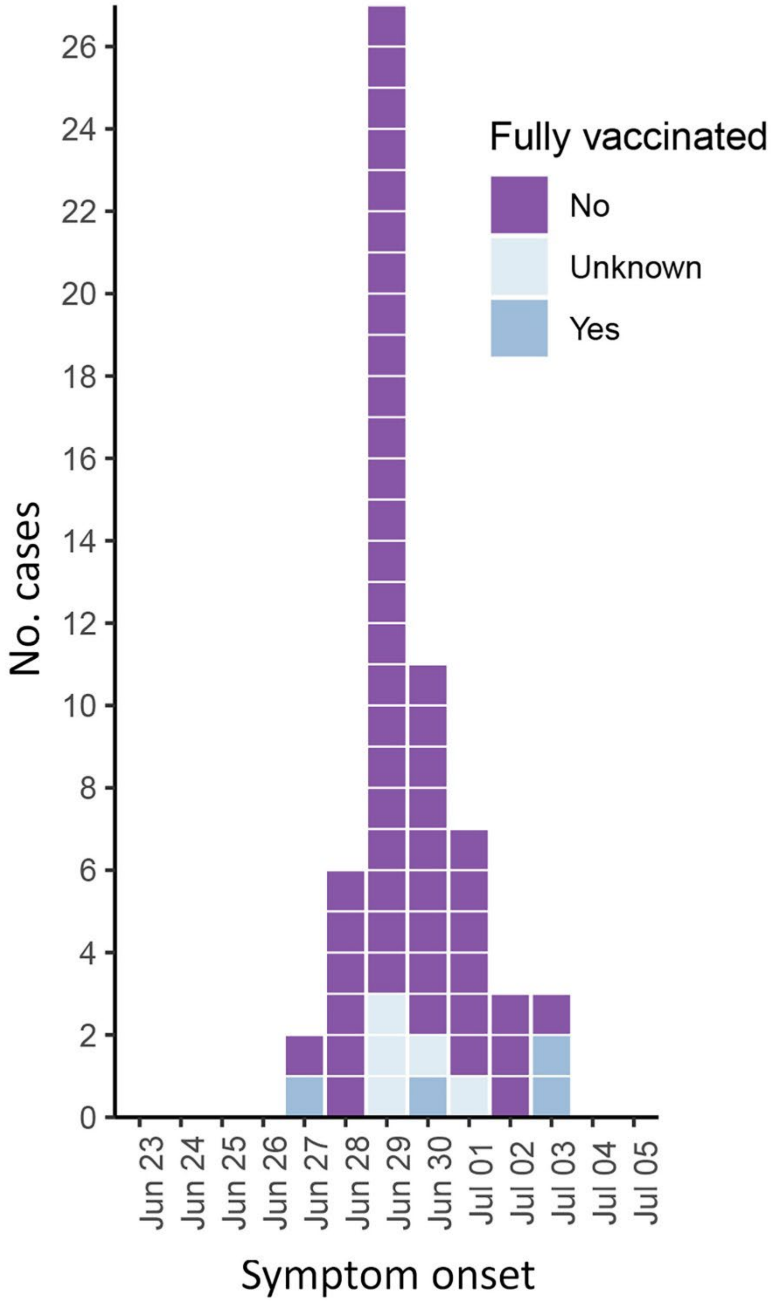

Appendix Figure 4. Epicurve of coronavirus disease cases related to a nightclub in central Amsterdam, the Netherlands, on June 26, 2021. Date of symptom onset is depicted, but for asymptomatic cases (n = 4), sampling date of positive test was used. For 1 case, date of symptom onset was missing and date of sampling and was omitted. Fully vaccinated is defined as 14 days after completion of vaccination series.

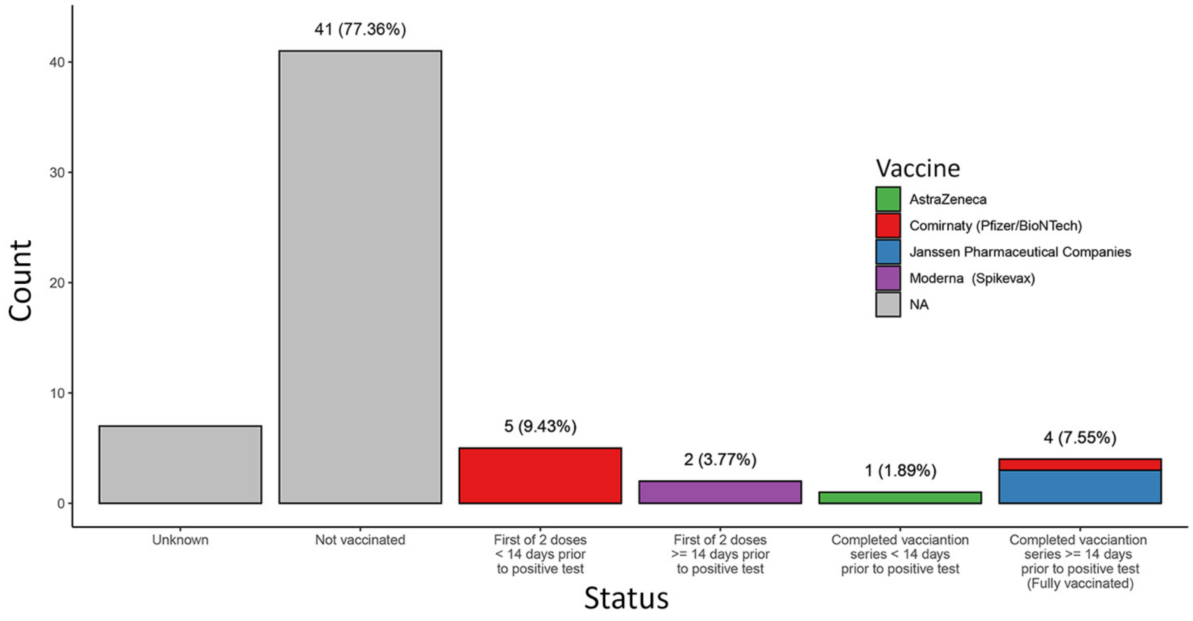

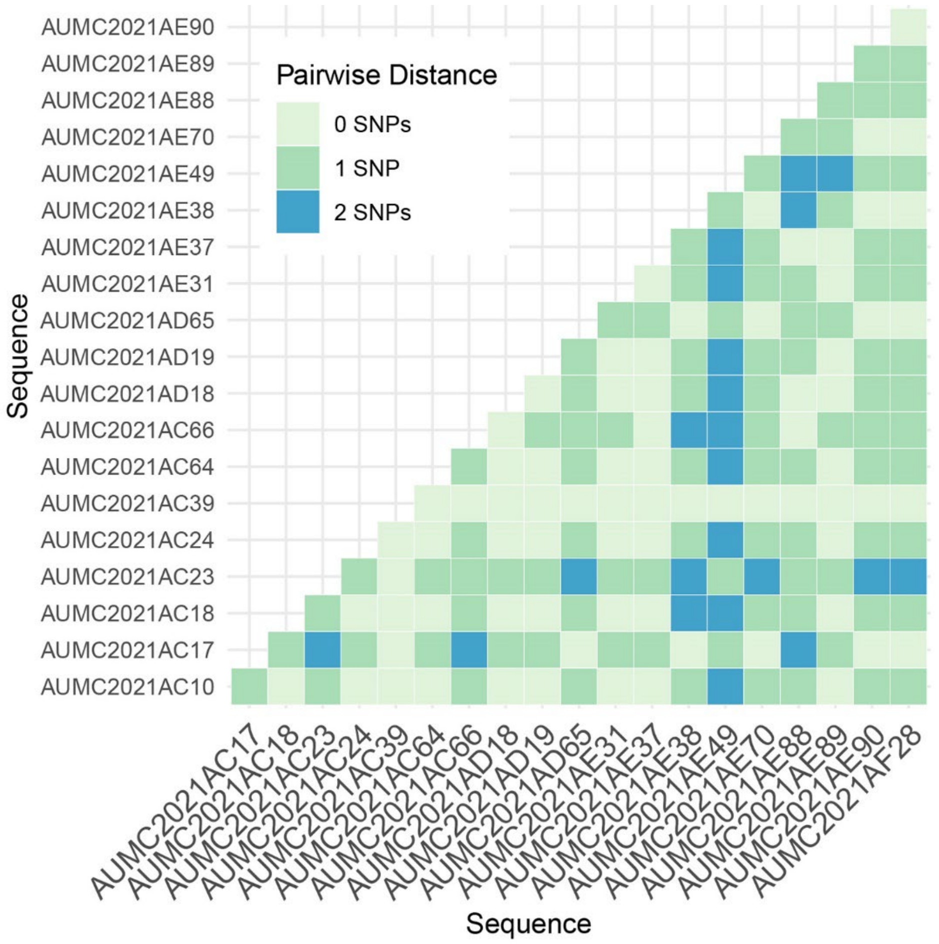

Appendix Figure 6. Pairwise distance matrix of all coronavirus disease sequences linked to event in nightclub, Amsterdam, the Netherlands, June 2021.

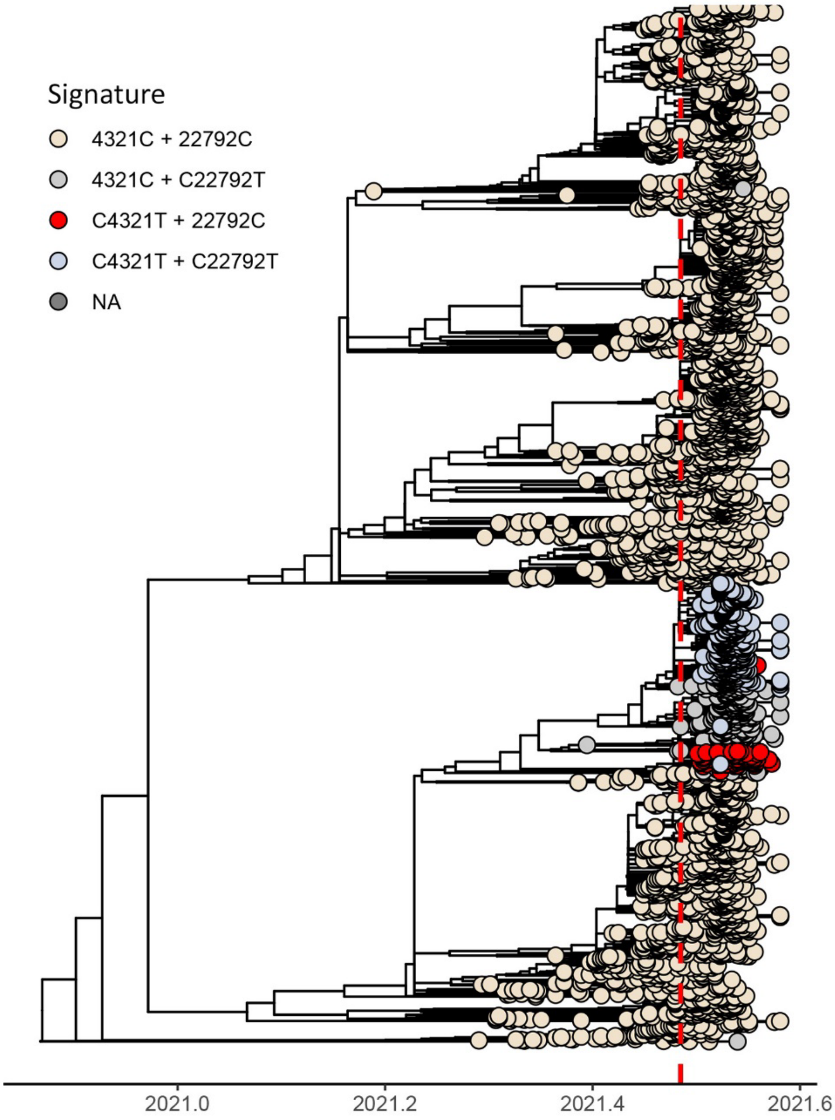

Appendix Figure 7. Time-resolved tree of dataset containing all Dutch severe acute respiratory syndrome coronavirus 2 Delta variant sequences available on GISAID on August 1, 2021, random surveillance samples from Amsterdam region, and samples from returning travelers to the Amsterdam region. Red dots denote samples with mutation signature observed in sequences linked to nightclub. Red dashed line indicates June 26, 2021, the date of the event at the nightclub.

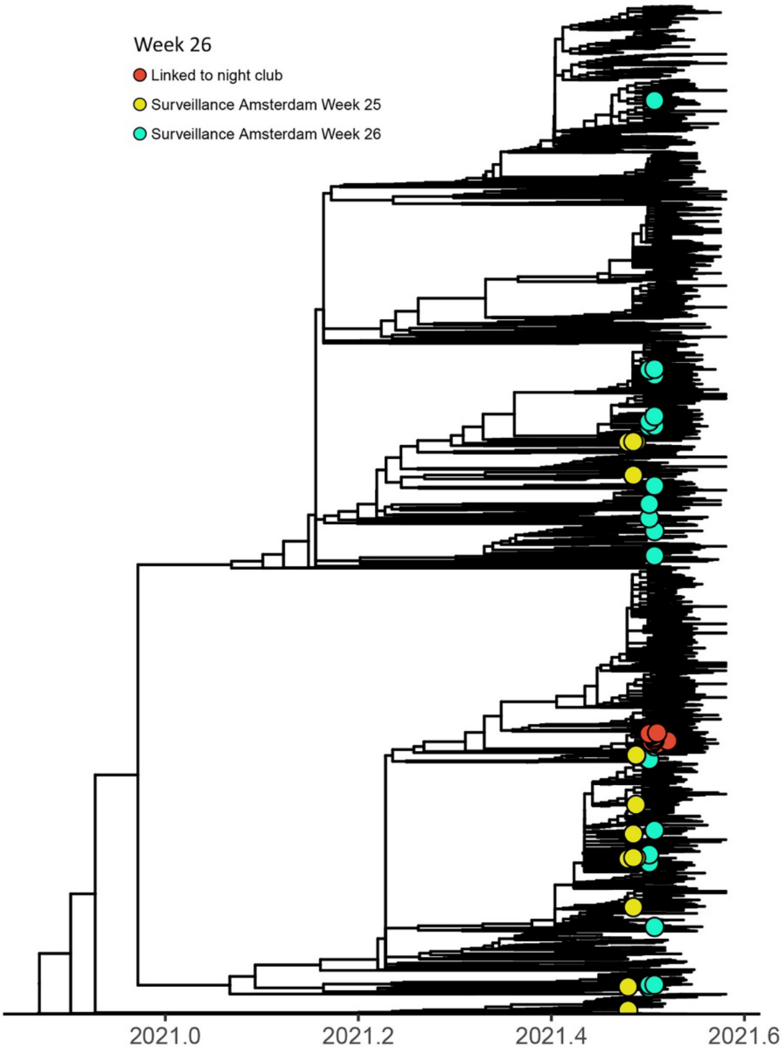

Appendix Figure 8. Time-resolved tree of dataset containing all Dutch severe acute respiratory syndrome coronavirus 2 Delta variant sequences available on GISAID on August 1, 2021, random surveillance samples from Amsterdam region, and samples from returning travelers to the Amsterdam region. Random surveillance samples from week 25 and week 26 (2021) are highlighted, together with sequences linked to the nightclub.

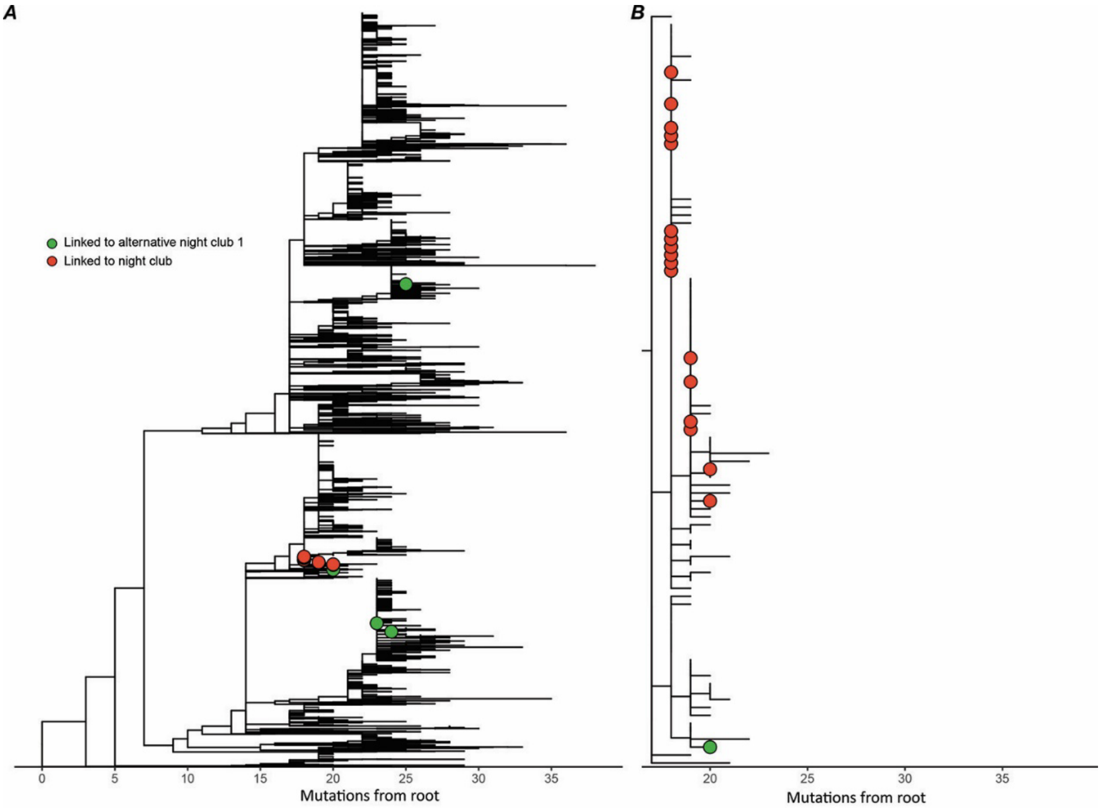

Appendix Figure 9. A) Phylogenetic tree containing all Dutch severe acute respiratory syndrome coronavirus 2 Delta variant sequences available on GISAID on August 1, 2021, random surveillance samples from Amsterdam region, samples from returning travelers to the Amsterdam region, and sequences linked to 2 additional nightclub events on June 26, 2021. Sequences linked to alternative nightclub 2 are not shown because they belonged to the B.1.1.7 lineage and were thus unrelated. B) Magnification of the clade containing the nightclub samples.
